# Stressors in the relatives of patients admitted to an intensive care unit

**DOI:** 10.5935/0103-507X.20160055

**Published:** 2016

**Authors:** Angélica Adam Barth, Bruna Dorfey Weigel, Claus Dieter Dummer, Kelly Campara Machado, Taís Montagner Tisott

**Affiliations:** 1 Academic Course of Medicine, Universidade de Santa Cruz do Sul - Santa Cruz do Sul (RS), Brazil.

**Keywords:** Stress, Family, Coma, Visits to patients, Critical care, Humanization of assistance, Intensive care units

## Abstract

**Objective:**

To identify and stratify the main stressors for the relatives of patients admitted to the adult intensive care unit of a teaching hospital.

**Methods:**

Cross-sectional descriptive study conducted with relatives of patients admitted to an intensive care unit from April to October 2014. The following materials were used: a questionnaire containing identification information and demographic data of the relatives, clinical data of the patients, and 25 stressors adapted from the Intensive Care Unit Environmental Stressor Scale. The degree of stress caused by each factor was determined on a scale of values from 1 to 4. The stressors were ranked based on the average score obtained.

**Results:**

The main cause of admission to the intensive care unit was clinical in 36 (52.2%) cases. The main stressors were the patient being in a state of coma (3.15 ± 1.23), the patient being unable to speak (3.15 ± 1.20), and the reason for admission (3.00 ± 1.27). After removing the 27 (39.1%) coma patients from the analysis, the main stressors for the relatives were the reason for admission (2.75 ± 1.354), seeing the patient in the intensive care unit (2.51 ± 1.227), and the patient being unable to speak (2.50 ± 1.269).

**Conclusion:**

Difficulties in communication and in the relationship with the patient admitted to the intensive care unit were identified as the main stressors by their relatives, with the state of coma being predominant. By contrast, the environment, work routines, and relationship between the relatives and intensive care unit team had the least impact as stressors.

## Abstract (pt)

**Objetivo:**

Identificar e estratificar os principais fatores estressores para os familiares de pacientes internados na unidade de terapia intensiva adulto de um hospital escola.

**Métodos:**

Estudo transversal descritivo com familiares de pacientes internados na unidade de terapia intensiva no período de abril a outubro de 2014. Foi utilizado questionário contendo identificação e dados demográficos dos familiares, dados clínicos dos pacientes, bem como 25 fatores estressores adaptados da Escala de Estressores em Unidade de Terapia Intensiva. O grau de estresse de cada fator foi determinado por uma escala de valores pontuando de 1 a 4. Os fatores estressores foram ranqueados conforme média dos pontos obtida.

**Resultados:**

A principal causa de admissão na unidade de terapia intensiva foi clínica em 36 (52,2%) casos. Os principais fatores estressores foram a presença do estado de coma (3,15 ± 1,23), o paciente não conseguir falar (3,15 ± 1,20) e o motivo da internação (3,00 ± 1,27). Quando retirados da análise os 27 (39,1%) pacientes em coma, os fatores de maior estresse para os familiares foram o motivo da internação (2,75 ± 1,354), ver o paciente na unidade de terapia intensiva (2,51 ± 1,227) e o paciente não conseguir falar (2,50 ± 1,269).

**Conclusão:**

A dificuldade na comunicação e na relação com o paciente internado na unidade de terapia intensiva foi apontada como os maiores estressores por seus familiares, com destaque para o estado de coma. Por outro lado, o ambiente, as rotinas de trabalho e a relação entre familiar e equipe da unidade de terapia intensiva tiveram menor impacto como fatores estressores.

## INTRODUCTION

The intensive care unit (ICU) is formed by a set of functionally grouped elements, intended to care for critically ill patients who require uninterrupted medical and nursing care, in addition to specialized human resources and equipment. Due to the intensive nature of this type of care, admission to an ICU is a stressful event, both for the patient and for his or her family, causing both physical and mental stress.^(1,2)^

The admission to an ICU generates a high degree of stress and anxiety to the family. The environment is perceived by them as an aggressive and threatening space because it evidences the risk of the patient dying. Consequently, the ICU environment can trigger behaviors and feelings such as doubt, helplessness, mental disorganization, inability to take action when faced with unexpected decisions, and other reactions, including depression or diseases caused by stress and anxiety.^(3,4)^

Previous studies show that relatives consider the appearance of the hospitalized patient; the need for mechanical ventilation; the presence of dressings, wires, and equipment; and the noise of the equipment and staff to be key stressors. In addition to these factors, the severity of the clinical picture, an altered level of consciousness, and difficulty and/or lack of communication were also identified as stressors.^(2,5,6)^

Anxiety, fear, and discomfort characterized by physiological and psychological changes are directly related to stress and occur when the individual is forced to face situations that are beyond his or her coping abilities.^(7)^ Novaes et al. compared stressors present in the ICU from the viewpoint of patients, relatives, and a multidisciplinary team; situations such as feeling pain, difficulty sleeping, and having tubes in the nose and/or mouth were most associated with the development of stress in the three groups.^(8,9)^

Visits by relatives is extremely important for the recovery of patients admitted to the ICU. For this reason, the Technical Regulation for Operation of Intensive Care Units (Resolution of Board of Directors [*Resolução da Diretoria Colegiada* - RDC] no. 7) was created, which specifies the minimum operating requirements of ICU. Although the RDC ensures rights to families, many ICU have strict routines that hinder the maintenance and strengthening of affective bonds between the patients and their relatives.^(10,11)^

Despite the existing knowledge about the different stressors in the ICU, most studies have focused on the stress experienced by patients as a result of their admission to the ICU. However, admission to the ICU also generates a high degree of stress and anxiety in the family. Thus, the present study sought to identify and stratify the main stressors as perceived by the relatives of patients admitted to the adult intensive care unit of a teaching hospital.

## METHODS

This cross-sectional descriptive study was conducted with relatives of patients admitted to the adult ICU of a medium-size teaching hospital located in the countryside of the state of Rio Grande do Sul, Brazil. The study was approved by the Research Ethics Committee of the *Universidade de Santa Cruz do Sul*, under number 33217914.1.0000.5343. At the time of the study, the ICU had 10 beds, with two isolation cubicles separated by walls, while the remaining were separated only by curtains. Each cubicle had one chair per patient, without a bathroom or individual television. No recreational activities were available. The patients were placed with their back to the external environment (west side), facing the nursing station, the prescription counter or the unit's corridor. The windows provided natural lighting, which was complemented by artificial lighting. The equipment with sound and light alarms were located at the head of the bed.

The health teams constantly performed different interventions in the patients, as part of routine care.

The ICU was a mixed unit, i.e., patients of both genders were admitted, which provided care for clinical, surgical or polytrauma patients, from the *Sistema Único de Saúde* (SUS), partner and private institutions. Eight of the ten beds were allocated for the SUS, and the remaining for partner and private institutions.

The hospital was a reference center in high-complexity cardiovascular care for the 13^th^ Regional Health District, and high-complexity in traumatology/orthopedic, elective surgeries and urgency for the municipalities that compose the 8^th^ and 13^th^ Regional Health Districts.

The unit performed the risk, severity, and prognostic stratification of their patients using the Acute Physiology and Chronic Health Disease Classification System II (APACHE II) and Sequential Organ Failure Assessment (SOFA) scores.

The study was conducted in the period from April to October 2014. The inclusion criteria were as follows: first-degree relatives (parents, siblings, or children) or spouse, older than 18 years of age, with the family member admitted to the ICU for over 24 hours, regardless of the reason for admission, belonging to any degree of risk stratification according to the scores used in the unit, who have visited the patient at least twice before completing the questionnaire. Identification and demographic data of the relatives, as well as clinical and hospitalization data of patients were collected. The visit time followed the criteria already established in the ICU routine, with two 30-minute periods, one in the morning and another in the afternoon.

To evaluate and stratify the stressors in ICU, the Intensive Care Unit Environmental Stressor Scale (ICUESS) translated and culturally adapted by Rosa et al. and validated by Ballard and Nastasy was used, which was originally composed of 40 items.^(12-14)^ In the present study, the ICUESS was adapted, resulting in a questionnaire with 25 items. Stressors were classified into four categories: environmental, patient-related, related to the interaction between the team and the family, and related to the visit or administrative routines. Every stressor was assigned a value of 1 to 4, with 1 being considered as not stressful, 2 as slightly stressful, 3 as stressful, and 4 as very stressful.

Only one relative per patient was interviewed. The means obtained for each of the stressors were ranked from highest to lowest. The data were analyzed using the Statistical Package for Social Science (SPSS), 17.0 software. Continuous variables were expressed as the means and standard deviations, and the categorical variables were expressed as absolute numbers and percentages. A simple percentage was used to characterize the sample, according to the clinical and sociodemographic variables; descriptive statistics and Student's *t*-test were applied for comparing the means. The level of significance was set as p < 0.05.

## RESULTS

During the study, 97 patients were admitted to the ICU; of these, only 69 relatives participated. The main reasons for exclusion were patients hospitalized for fewer than 24 hours and relatives who made only one visit to the ICU. The male gender was predominant, with 52 (75.4%) cases. The age of the relatives was 46.46 ± 1.10 years. As for the degree of kinship, 29 (42%) were sons/daughters, 13 (13%) were spouses, 12 (17.4%) were fathers/mothers, and 10 (14.5%) were brothers/sisters. Thirty-nine (56.5%) relatives had some type of paid occupation, while the remaining 24 (34.7%) were retired or not working. Regarding education, 31 (44.9%) had completed elementary school, 19 (27.5%) had completed secondary school, seven (10.1%) had completed higher education, and five (7.2%) were illiterate. The most prevalent religions were Catholic and Protestant, with 46 (73%) and 11 (17.5%) individuals, respectively. Table 1 summarizes the sociodemographic characteristics of the relatives who participated in the study.

**Table 1:** Sociodemographic characteristics of the relatives

Variables	Frequency
Sex	
Male	52 (75.4)
Age (years)	46.46 ± 11.10
Education level	
Illiterate	5 (7.2)
Completed elementary school	31 (44.9)
Completed secondary school	19 (27.5)
Higher education	7 (10.1)
Occupation	
Working	39 (56.5)
Not working	9 (13)
Retired	15 (21.7)
Degree of kinship	
Spouse	13 (18.8)
Father/mother	12 (17.4)
Children	29 (42)
Sibling	10 (14.5)
Religion	
Catholic	46 (66.7)
Protestant	11 (15.9)
Spiritist	1 (1.4)
Jehovah’s witness	1 (1.4)
Other	4 (5.8)

Results expressed as the number (%) and mean ± standard deviation.

In 36 (52.2%) cases, the reason for patient admission to the ICU was clinical. The mean length of stay was 3.14 ± 4.08 days, and the number of visits made by the relatives was 3.62 ± 4.36. Thirty-four (54%) patients did not require mechanical ventilation. The main stressors of the relatives were the patient being in a state of coma (3.15 ± 1.23), the patient being unable to speak (3.15 ± 1.20), and the reason for admission (3.00 ± 1.27). As the first two stressors had the same mean value, the one with the larger standard deviation was ranked as first. The other stressors are described in table 2.

**Table 2:** Stressors evaluated by relatives of patients admitted to the ICU

Stressors	Mean ± SD
Patient	
State of coma	3.15 ± 1.231
Inability to speak	3.15 ± 1.202
Reason for admission	3.00 ± 1.279
Seeing the patient in the ICU	2.81 ± 1.240
Being tied to a tube	2.71 ± 1.349
Being tied/restrained	2.48 ± 1.417
Length of stay	1.90 ± 1.214
Being uncovered	1.87 ± 1.180
Lack of clothing	1.56 ± 1.040
Environment	
Equipment around the patient	1.68 ± 1.022
Noise from the equipment	1.37 ± 0.771
ICU environment	1.33 ± 0.700
Environmental noise	1.21 ± 0.612
Lighting	1.16 ± 0.474
ICU smells	1.10 ± 0.462
Number of patients in the ICU	1.25 ± 0.673
Team	
Not knowing the team	1.49 ± 0.954
Contact with the ICU physician	1.27 ± 0.795
Information given	1.23 ± 0.710
Relationship with the ICU team	1.17 ± 0.513
Visit	
Visiting schedule	1.75 ± 1.035
Not having a companion	1.76 ± 1.053
Delay in the visits	1.60 ± 1.025
Visit time	1.57 ± 0.886

SD - standard deviation; ICU - intensive care unit.

Subsequently, the stressors were evaluated considering the presence or absence of a state of coma in the admitted patients. Table 3 presents only the means of stressors with a statistically significant difference between those two groups.

**Table 3:** Stressors evaluated according to the presence or absence of coma

Stressors	Presence of coma	Absence of coma	p-value
Reason for admission	3.46	2.75	0.017
Inability to speak	3.43	2.50	0.038
Seeing the patient in the ICU	3.33	2.51	0.006
Length of stay	2.30	1.61	0.032
Being tied to a tube	3.23	1.88	0.001
Being uncovered	2.28	1.61	0.040
Equipment noise	1.78	1.10	0.003
Equipment around the patient	2.11	1.41	0.014

ICU - intensive care unit. Only the means of stressors with statistically significant difference are presented.

## DISCUSSION

The technological evolution, and the increased ability and experience in managing critically ill patients, as well as the dissemination of knowledge by the lay population, have led to changes in the approach of intensive care professionals. The exclusively technical focus has been questioned from an ethical and humanitarian viewpoint, and an approach focused on the interests of the patient has been shown to be feasible and effective. Recently, greater attention has been given to the assistance of families.^(2,15-17)^

Despite the reduced sample size, the present study may provide important information about stressors in ICU. Most of the relatives were male, unlike what was observed by Costa et al. and Santos et al., who found women to be the present and participative relative.^(2,11)^ In the visits to the ICU, the predominant degree of kinship was son/daughter (42%), with a mean age of 46.4 years. In the study by Piccini et al., conducted in the same ICU during the first half of 2015, the mean age of the patients admitted was 56 ± 15.04 years, and 69.4% were female, which are findings similar to those in this study.^(18)^

Most relatives had some type of paid occupation (56.5%), which might have created difficulties for making the hospital visits, as they took place in the morning and afternoon. Such a fact can be represented by the low score of the stressors "having no companion" and "visit time", given the need for the relative to work.

Notably, the study was restricted to the early assessment of family stressors, in the first days of admission in the ICU. There was no other evaluation during the course of hospitalization. This can be considered a limitation of the study, and it is believed that a longer stay in the ICU and a larger number of visits by relatives might have caused certain stressors (which were not observed before or did not appear to be of great relevance) to have become more relevant over time. Heidemann et al. assessed the main stressors in patients admitted to a coronary unit by applying the ICUESS in the first, second, and third days of hospitalization, and the medians were 67.5, 60.5, and 59.5, respectively. These results show a reduction in the perceived stress in patients over the first three days of hospitalization, although no statistically significant difference was observed between the values.^(19)^

In the study by Novaes et al., the stressor "seeing family and friends only a few minutes a day" was more relevant for the relatives, ranking in the 9^th^ position, than for the patients admitted to the ICU, occupying the 12^th^ position. Thus, the family overestimates its presence at the patient's bedside, while the latter gives greater relevance to his or her recovery, as long as the family can be present at flexible times.^(9)^

Regarding education, 44.9% had completed elementary school, and only 10.1% had completed higher education. The most prevalent religion was Catholic, at 73%. These last two findings are in agreement with the data from the 2010 census of the Brazilian Institute of Geography and Statistics (*Instituto Brasileiro de Geografia e Estatística* - IBGE), whereby only 7.9% of Brazilians have higher education and 64.6% of Brazilians are Catholic. Notably, socioeconomic and cultural conditions differentially affect how patients and their relatives perceive the ICU and critical illness. Such variables, along with ethnic and religious differences, can lead to perceive difficulties and different reactions, or even to conflicts.^(2,17,20,21)^

In this study, the factors that contributed the most to stress for the relative were the state of coma, inability to speak and the reason for admission to the ICU. The relatives considered changes in the level of consciousness and difficulty and/or lack of communication to be main stressors because these factors preclude the patient from being able to make decisions, thus transferring such responsibilities to the family.^(2,5)^ Patients in a coma need respiratory support in most cases, which prevents the patient from being able to speak and interact with their relatives. This generates a feeling of helplessness in the relatives. The possibility of dialogue favors a decrease in the anxiety of both patients and relatives.^(2,5)^

A differential in the study was the profile of the admitted patients, which differed from that described in previous studies,^(8,9)^ in which the prerequisite for participation of the relative in the study was that the patient was conscious, lucid, and breathing spontaneously. Although for most study participants their relative was not in a coma, this factor was what generated the greatest stress among the relatives interviewed. Moreover, not being in a coma may have contributed to items as "being unable to speak" and "being tied to the tube" having a lower score.

Studies^(4,9)^ have assessed the stress of patients from their perspective, that of relatives, and that of the healthcare team, revealing that "feeling pain" is considered the main stressor in all groups. Thus, the healthcare team should pay close attention to signs of pain, aiming to do the best they can to relieve the pain of the patient.^(9)^

The reason for admission is also among the main stressors. In this study, most patients admitted to the ICU were so for clinical reasons (52.2%). Possibly, this finding was related to the demographic transition occurring in Brazil, in which elderly patients present chronic and complex diseases, to the detriment of surgical cases. The study by Neves et al. that assessed the degree of satisfaction of relatives of patients admitted to ICUs also found similar results regarding the type of admission, with hospitalization for clinical reasons leading to lower degree of satisfaction of the relative compared to those admitted for surgical reasons, likely because the former are chronic patients with a more severe condition.^(22)^

The score for the stressors related to the nursing team and the ICU physician were lower. This highlights the trust placed on the ICU team, as well as on the information provided regarding the condition and the evolution of the patient. It is known that most needs considered important by relatives are dependent on the initiative of the professionals, who must seek to better the relationship with the family, clarifying the chances for improvement and properly informing them about the patient's evolution. This information should be provided by the professional daily-responding to questions with honesty, clarifying who are the professionals involved in the care of the patient, and ensuring that the treatment adopted is the best possible one. Such information should be easy to understand.^(23)^ Gaeeni et al. revealed that relatives want to be supported by the ICU team by being present and available, providing information about the clinical conditions and the result of the treatments of their family member. The participants also agreed on the importance of providing information about the reason for admission to the ICU and the equipment in the unit.^(24)^

The environmental factors, such as smells, noises, and lighting, generated less stress in this study. These findings may be related to the fact that ICUs must follow the guidelines established by the National Health Surveillance Agency (*Agência Nacional de Vigilância Sanitária* - ANVISA).^(10)^ The RDC no. 7, from February 24, 2010, is an example of such guidelines. This resolution aims to establish minimum operating standards for ICUs, aiming to reduce risks for patients, visitors, professionals, and the environment. Furthermore, Stricker et al.^(25)^ considered the visit time a limiting factor in the perception of the factors related to the ICU (the inclusion criteria was a minimum of two visits of 10 minutes each, which is not a lot of time for evaluating the patient care and the surrounding environment). In the ICU studied here, the visit time was limited to 30 minutes, twice a day. Similarly, Costa et al.^(2)^ also reported the ICU environment as a weak stressor. In turn, Lemos and Rossi found two distinct positions. On one hand, the factors related to the environment, cleanliness, and organization generated among relatives a perception of the ICU as a specialized environment for the care and recovery of critically ill patients, providing security and peace of mind to the family. On the other hand, factors such as noise, excessive movement, and excess light are considered nuisances that help to hinder the care provided.^(6)^

The measures to be taken to reduce stress in relatives include counseling and psychological support, especially when loved ones are at risk of dying. In addition, follow-up is recommended because symptoms of anxiety, depression, and PTSD persist over time in family members; in patients, these symptoms last 3 months.^(26)^

Open visits to the ICU, which allows relatives to visit the patients at any time of day, is another factor impacting the stress reduction in relatives, according to a study published in Plos One.^(26)^ The *Sociedade Paulista de Terapia Intensiva* (SOPATI) argues that a more flexible policy of visitation to patients admitted to ICUs helps families face this situation, satisfying their great need to be near the patients. The benefit of open visits was reported in a study conducted in the ICU of the *Hospital Sírio-Libanês* in 2015, in which of the 471 families interviewed, 33% presented symptoms of anxiety and 18% presented symptoms of depression.^(27)^

However, although some stress factors for relatives can be addressed, other factors, such as the state of coma, indicated in our study as the main stressor, cannot be modified because it is inherent to the clinical condition of the patient. To minimize the impact of this situation on family members, measures such as humanized care can mitigate the emotional impact.

Thus, the care of the family of the ICU patient must direct the team of professionals toward a comprehensive approach, including sensitivity and attention. The healthcare professionals must show respect and evaluate the family based on reliable clinical and interpersonal criteria and judgments,^(28)^ even considering the diversities and peculiarities of ICU. Studies with the purpose of identifying stressors should be encouraged. Such investigations would enable developing individualized strategies for minimizing the impact of these stressors on this fragile population: the relatives of ICU patients.

## CONCLUSION

The demographic profile of the relatives of patients admitted in the intensive care unit showed a predominance of middle-aged workers, males, Catholics, with completed elementary education. The stressors of greater impact according to the perception of the relatives in the study were the state of coma and difficulties in the communication between relative and patient. Such factors do not favor the interaction of families with the unconscious patient, and thus, it is impossible for the relative to stimulate the patient in his or her recovery. By contrast, the environment, the intensive care unit routines, and the relationship between family and intensive care team had the lowest impact as stressors for the relatives. Individualized strategies to minimize and prevent the impacts of stressors in the intensive care unit, as well as reception and care measures focusing on the families of hospitalized patients, must be developed.
